# Caplacizumab-Enabled Treatment of Immune-Mediated Thrombotic Thrombocytopenic Purpura Without Plasma Exchange: Evidence, Patient Selection, and Practical Considerations

**DOI:** 10.1177/10760296261468955

**Published:** 2026-08-03

**Authors:** Nabeel Qasem, Yousef Al-Asa’d, Mohammed Abdulgayoom, Awni Alshurafa, Mohamed Yassin

**Affiliations:** 1 Department of Hematology, 62850 National Center for Cancer Care and Research, Hamad Medical Corporation, Doha, Qatar

**Keywords:** immune-mediated thrombotic thrombocytopenic purpura, caplacizumab, therapeutic plasma exchange, ADAMTS13, von Willebrand factor, rituximab, plasma exchange-free treatment

## Abstract

**Background:**

Immune-mediated thrombotic thrombocytopenic purpura (iTTP) is a rare, life-threatening autoimmune condition driven by severe ADAMTS13 deficiency. Therapeutic plasma exchange (TPE) is the traditional cornerstone of acute therapy, as it clears autoantibodies and replenishes functional ADAMTS13. However, caplacizumab—an anti-von Willebrand factor nanobody that rapidly blocks platelet-von Willebrand factor interactions—has raised the possibility of safely omitting TPE in carefully selected patients.

**Objective:**

To review the biological rationale, current clinical evidence, patient-selection criteria, safety profile, and practical implementation of utilizing caplacizumab for iTTP treatment without relying on plasma exchange.

**Methods:**

A comprehensive narrative review was conducted. It analyzed published case reports, case series, registry data, comparative cohort studies, and guideline-relevant literature focusing on TPE-free or TPE-sparing caplacizumab-based regimens for iTTP.

**Results:**

Evidence has evolved from isolated, necessity-driven cases to robust multicenter comparative data. Caplacizumab-based, TPE-free treatment combined with immunosuppression consistently drives rapid platelet recovery, typically within three to five days. Large comparative cohorts demonstrate that clinical response, exacerbation rates, refractoriness, and mortality are similar to conventional TPE-based therapies. Furthermore, the TPE-free approach offers the benefits of shorter hospital stays and reduced intensive care utilization. However, a subset of patients with inadequate initial platelet responses or complex comorbidities still required rescue TPE.

**Conclusion:**

TPE-free caplacizumab therapy is a promising, highly targeted strategy, though it should not replace standard therapy outside experienced centers. Safe implementation demands confirmed iTTP, clinical stability, immediate caplacizumab availability, rigorous ADAMTS13 monitoring, adequate immunosuppression, and predefined thresholds for rescue TPE.

## 1. Introduction: Why Consider Plasma Exchange-Free Treatment?

Immune-mediated thrombotic thrombocytopenic purpura (iTTP) is a rare and potentially fatal autoimmune thrombotic microangiopathy caused by severe functional deficiency of ADAMTS13 due to inhibitory autoantibodies. Severe ADAMTS13 deficiency permits persistence of ultra-large von Willebrand factor (VWF) multimers, which promote pathological platelet adhesion, disseminated microvascular platelet-rich thrombosis, microangiopathic haemolytic anaemia, thrombocytopenia, and variable organ ischaemia, particularly involving the brain, heart, kidneys, and gastrointestinal tract.^1^

For more than three decades, therapeutic plasma exchange (TPE) has been the central acute intervention in iTTP. The landmark trial by Rock and colleagues established the superiority of plasma exchange over plasma infusion and transformed a historically highly fatal disease into a treatable emergency.^2^ The biological appeal of TPE is broad: it removes circulating anti-ADAMTS13 antibodies and ultra-large VWF multimers while replacing deficient ADAMTS13 with donor plasma. Current treatment frameworks therefore continue to emphasize early recognition, urgent initiation of TPE, corticosteroids, B-cell-directed immunosuppression in appropriate patients, and caplacizumab where available.^3,4^

Despite its benefit, TPE is invasive, resource-intensive, and not risk-free. It requires vascular access, specialised apheresis expertise, donor plasma exposure, daily procedure capacity, and close monitoring. Complications include catheter-related bleeding, thrombosis or infection; allergic and anaphylactic reactions to plasma; citrate-related hypocalcaemia; haemodynamic instability; and substantial logistical burden.^2,5^ These limitations are particularly relevant when TPE is refused because of religious beliefs, contraindicated because of severe plasma reactions, technically difficult because of venous access limitations, or unavailable within the required timeframe.

The development of caplacizumab has changed the therapeutic logic of acute iTTP. Caplacizumab is a humanised bivalent nanobody targeting the A1 domain of VWF. By preventing VWF binding to platelet glycoprotein Ib-IX-V, it rapidly interrupts microvascular platelet aggregation independently of ADAMTS13 recovery.^4^ An updated systematic review and meta-analysis of 13 studies encompassing 2,956 iTTP patients subsequently confirmed these benefits, demonstrating significant reductions in mortality (risk ratio [RR] 0.29; 95% confidence interval [CI] 0.19–0.46), exacerbations (RR 0.27; 95% CI 0.13–0.58), and TPE requirements, with no significant increase in major bleeding or thrombosis.^6^ This mechanism raises an important clinical question: in selected patients, can caplacizumab provide sufficient acute anti-thrombotic control to allow safe omission of TPE while immunosuppression restores ADAMTS13 activity? This review synthesises the rationale, published evidence, patient-selection principles, safety issues, and practical requirements for this emerging approach.

**Figure 1.**
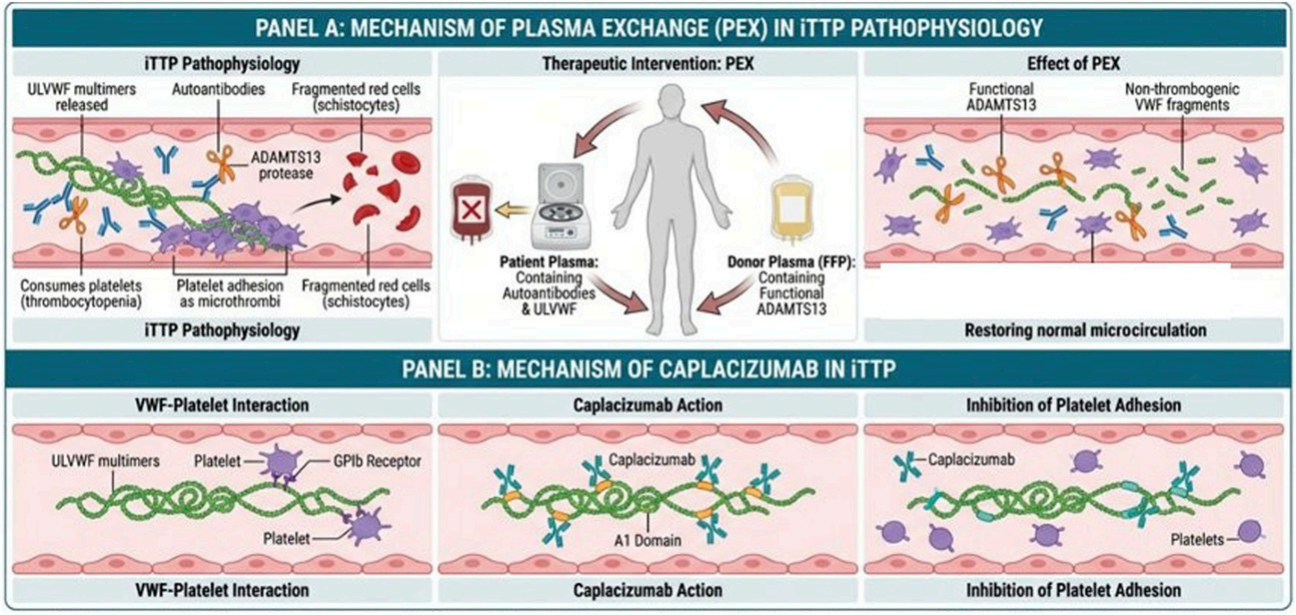
Mechanisms of action of therapeutic plasma exchange (PEX) and caplacizumab in immune-mediated thrombotic thrombocytopenic purpura (TTP). Panel A illustrates the pathophysiology of ITTP, in which inhibitory autoantibodies against ADAMTS13 lead to the accumulation of ultra-large von Willebrand factor (ULVWF) multimers, promoting pathological platelet adhesion, microvascular thrombosis, and consumptive thrombocytopenia. TPE addresses this by simultaneously removing autoantibodies and ULVWF multimers while replenishing functional ADAMTS13 from donor plasma, thereby restoring normal microcirculation. Panel B illustrates the mechanism of caplacizumab, a humanized bivalent nanobody targeting the A1 domain of VWF and blocking its interaction with the platelet glycoprotein lb (GPIb) receptor, rapidly halting microvascular thrombus formation independently of ADAMTS13 activity

Figure 1. Treatment Mechanisms in iTTP. Panel A: Pathophysiology of iTTP and the role of therapeutic plasma exchange (PEX). PEX restores normal microcirculation by removing ADAMTS13 autoantibodies and ultra-large von Willebrand factor (ULVWF) multimers while replenishing functional ADAMTS13 from donor plasma. Panel B: Mechanism of action for caplacizumab. This nanobody binds to the A1 domain of VWF, directly blocking platelet adhesion to the GPIb receptor and halting microthrombus formation independently of ADAMTS13 levels.

## 2. Methods and Scope of the Review

This narrative review focuses on published experience with caplacizumab-enabled management of iTTP without first-line TPE. The evidence base includes case reports, small series, registry-derived observations, retrospective cohort data, guideline-relevant literature, and clinical trial information. Because the available literature is heterogeneous and largely non-randomised, the review does not attempt quantitative synthesis. Instead, it organises the evidence around clinically relevant questions: biological plausibility, reported outcomes, candidate selection, treatment components, ADAMTS13 recovery, safety, implementation, and unresolved questions.

The term “plasma exchange-free treatment” is used throughout to describe treatment strategies in which TPE is deliberately omitted during the acute episode. This should be distinguished from “plasma exchange-sparing” approaches, in which caplacizumab shortens the duration or number of TPE procedures but does not eliminate them.

## 3. Biological Rationale for a Plasma Exchange-Free Strategy

### 3.1. Pathophysiology of Immune-Mediated TTP

The central lesion in iTTP is severe ADAMTS13 deficiency, usually defined as activity below 10%, caused by autoantibody-mediated inhibition, accelerated clearance, or both.^1^ In the absence of adequate ADAMTS13 activity, ultra-large VWF multimers persist and bind platelets under high shear stress, producing disseminated platelet-rich microthrombi. The resulting clinical syndrome is characterised by thrombocytopenia, microangiopathic haemolytic anaemia, elevated lactate dehydrogenase, schistocytes on blood film, and organ injury of variable severity. Because organ injury can progress rapidly, treatment is usually initiated on high clinical suspicion while confirmatory ADAMTS13 testing is pending.^1,3^

### 3.2. What Therapeutic Plasma Exchange Contributes

TPE acts on several levels. It removes pathogenic anti-ADAMTS13 antibodies, clears ultra-large VWF multimers, and replaces functional ADAMTS13.^2,3^ These combined actions explain why TPE became the historical standard of care. However, TPE does not selectively remove only pathogenic antibodies, and repeated donor plasma exposure may have immunological consequences. Moreover, TPE does not directly suppress ongoing autoantibody production, which is why immunosuppression is an essential component of modern management.^3^

### 3.3. What Caplacizumab Replaces - And What It Does Not Replace

Caplacizumab addresses the immediate downstream consequence of severe ADAMTS13 deficiency by blocking VWF-mediated platelet adhesion.^4^ This rapid effect is mechanistically attractive in acute iTTP because platelet-VWF interaction is the final common pathway of microvascular thrombosis. In this sense, caplacizumab can partially substitute for the acute haemostatic effect of TPE by interrupting microthrombus propagation.^4,5^ However, caplacizumab does not remove anti-ADAMTS13 antibodies, replenish ADAMTS13, or correct the autoimmune basis of disease. Its protective effect persists only while the drug is present. Therefore, plasma exchange-free treatment should not be conceptualised as caplacizumab monotherapy; it is a combined strategy that requires effective immunosuppression and close ADAMTS13-guided monitoring.

### 3.4. Why Immunosuppression Remains Essential

Corticosteroids and B-cell-directed therapy, particularly rituximab, remain central to durable disease control.^3^ The purpose of immunosuppression is to reduce anti-ADAMTS13 antibody production and permit immunological remission. Without ADAMTS13 recovery, patients remain biologically vulnerable to exacerbation or relapse once caplacizumab is discontinued.^7,8^ This point is especially important in plasma exchange-free strategies, where the clinician intentionally omits TPE-mediated antibody removal and ADAMTS13 replacement. Adequate and timely immunosuppression is therefore a core safety pillar of this approach.

## 4. Published Clinical Experience With Plasma Exchange-Free Treatment

### 4.1. Early Necessity-Driven Cases: Plasma Refusal and Contraindication

Before caplacizumab, plasma exchange-free management was largely restricted to exceptional circumstances, most often Jehovah’s Witness patients refusing blood products. Reports from this era showed that survival without plasma could occur, but platelet recovery was slower and ADAMTS13 recovery was inconsistent.^9^ Caplacizumab changed this context by offering immediate pharmacological inhibition of VWF-platelet interaction without donor plasma exposure.^4,10^

Chander and colleagues reported one of the first caplacizumab-treated iTTP cases without TPE in a Jehovah’s Witness patient who refused plasma exchange.^10^ After deterioration on glucocorticoids, rituximab, and adjunctive therapy, caplacizumab was initiated through compassionate use and was followed by rapid platelet recovery and neurological improvement. This case provided proof-of-concept that pharmacological VWF blockade could rescue acute microvascular thrombosis when TPE was not acceptable.

Subsequent cases extended this experience. Warr described successful treatment of a Jehovah’s Witness patient with caplacizumab, prednisone, rituximab, and Koate-DVI, a plasma-derived factor VIII concentrate containing ADAMTS13.^11^ Cardesa-Salzmann and colleagues reported COVID-19-associated iTTP in a Jehovah’s Witness adolescent treated with caplacizumab, methylprednisolone, and rituximab; extracorporeal immunoadsorption was later used to facilitate ADAMTS13 recovery.^12^ Irani and colleagues described caplacizumab use in a patient with anaphylaxis to fresh-frozen plasma, supporting its potential value when TPE is unsafe because of severe plasma reactions.^13^

### 4.2. Patient Preference and Shared Decision-Making

Sukumar and colleagues reported a relapsing iTTP patient who wished to avoid hospitalisation, TPE, and corticosteroids.^14^ After shared decision-making, the patient received outpatient caplacizumab with subsequent rituximab and achieved ADAMTS13 recovery. This case is important because it moved the discussion beyond absolute contraindication or religious refusal. It suggested that, in highly selected and reliable patients with early relapse, careful shared decision-making may justify TPE omission when the clinical situation is stable and monitoring can be assured.

Shared decision-making should not be interpreted as patient preference alone overriding standard care. Rather, it requires transparent explanation of uncertainty, careful assessment of disease severity, explicit discussion of rescue TPE, and documentation of the monitoring plan.^14^ In first-presentation iTTP, diagnostic uncertainty is greater and the threshold for standard TPE-based treatment should remain low.

### 4.3. Relapsed Immune-Mediated TTP Treated Without Plasma Exchange

Relapsed iTTP may be particularly suitable for carefully selected plasma exchange-free treatment. Patients with known iTTP often present earlier, may already have an established ADAMTS13 monitoring pathway, and may be familiar with caplacizumab self-administration. Völker and colleagues reported real-world caplacizumab experience across German centres, including patients managed without TPE at presentation or during relapse/exacerbation, with platelet normalisation within several days.^15^ Capecchi and colleagues subsequently reported three relapsed iTTP patients managed without TPE using caplacizumab and individualised immunosuppression.^16^ These reports support the concept that early relapse, especially when mild and promptly recognised, may be an appropriate clinical context for TPE-free management.

A recent case report also described relapsing TTP managed without TPE using early caplacizumab, corticosteroids, and obinutuzumab, a second-generation anti-CD20 monoclonal antibody.^17^ Although this remains a single-patient experience, it reinforces the principle that effective B-cell-directed therapy may be relevant when TPE is omitted, particularly in patients with relapsed disease or prior rituximab exposure.

### 4.4. Multicentre Comparative Evidence

The strongest evidence to date comes from Kühne and colleagues, who reported a retrospective multicentre cohort comparing 42 patients treated without TPE with 59 patients treated with standard TPE-based management.^5^ Median time to platelet count normalisation was not significantly different between the groups. Clinical response, exacerbation, refractoriness, and mortality were also comparable. The plasma exchange-free cohort had shorter hospitalisation and lower intensive care utilisation. Four patients required rescue TPE after failing to demonstrate adequate early response to caplacizumab; these patients had identifiable comorbidities or confounding factors that likely impaired or obscured the platelet response.

This study is clinically influential because it shifted the field from isolated anecdote to comparative observational evidence. It also introduced the most practical decision point: early platelet trajectory after the first intravenous caplacizumab dose.^5^ Rather than relying only on baseline platelet count or disease label, this response-guided approach uses immediate biological feedback to decide whether TPE can remain withheld.

### 4.5. Summary of Clinical Outcomes Across Published Reports

Across reported experiences, caplacizumab-based TPE-free treatment has generally achieved platelet recovery within approximately three to five days when patients were appropriately selected and received adequate immunosuppression.^5,10-12,14-16^ The most common clinical contexts were plasma refusal, plasma allergy or procedural barriers, patient preference after shared decision-making, and relapsed iTTP recognised early.^10-16^ The main recurring safety concern was bleeding related to caplacizumab, especially with concomitant antiplatelet or anticoagulant therapy.^5,16^ The main recurring efficacy concern was inadequate ADAMTS13 recovery or premature discontinuation of caplacizumab before immunological remission.^5,7,8,16^ These findings are corroborated by an updated meta-analysis of 2,956 patients across 13 studies, which confirmed that early initiation of caplacizumab (within three days of diagnosis or plasma exchange commencement) was associated with a significantly shorter hospital stay (mean difference −8.6 days; 95% CI −12.4 to −4.8) compared with delayed initiation.^6^

Table 1 presents a Summary of published experiences using caplacizumab combined with immunosuppression to treat iTTP without therapeutic plasma exchange (TPE). It outlines specific clinical contexts (e.g., relapsing disease or plasma refusal), treatment regimens, clinical outcomes like platelet recovery, and key lessons regarding the safety and feasibility of TPE-free management.

**Table 1.** Selected Published Experience With Caplacizumab-Enabled Treatment Without Plasma Exchange

Study	Clinical context	Treatment approach	Key outcome	Main lesson
Chander et al.^10^	Jehovah’s Witness patient refusing plasma exchange	Caplacizumab with glucocorticoids and rituximab after clinical deterioration	Rapid platelet and neurological improvement	Proof-of-concept that VWF blockade can control acute disease when TPE is not possible
Sukumar et al.^14^	Relapsing iTTP; patient preference to avoid hospitalisation/TPE	Outpatient caplacizumab with rituximab	Platelet recovery and ADAMTS13 remission	Shared decision-making may be reasonable in carefully selected, reliable relapse patients
Völker et al.^15^	Real-world caplacizumab cohort including TPE-free episodes	Caplacizumab-based treatment with immunosuppression	Platelet normalisation within several days in reported TPE-free cases	Early recognition and prompt caplacizumab may permit TPE avoidance in selected episodes
Warr^11^	Jehovah’s Witness patient with severe iTTP	Caplacizumab, prednisone, rituximab, and Koate-DVI	Clinical and laboratory remission	Adjunctive ADAMTS13-containing products may be considered when plasma is refused
Cardesa-Salzmann et al.^12^	Jehovah’s Witness adolescent with COVID-19-associated iTTP	Caplacizumab, methylprednisolone, rituximab, and later immunoadsorption	Clinical response with subsequent ADAMTS13 recovery	Immunoadsorption may be an adjunct where ADAMTS13 recovery is delayed and TPE is unacceptable
Capecchi et al.^16^	Relapsed iTTP	Caplacizumab with individualised immunosuppression	Clinical responses within days; one major bleeding event	Relapsed disease may be suitable, but bleeding risk and ADAMTS13 kinetics require caution
Kühne et al.^5^	Multicentre retrospective comparison of TPE-free versus TPE-treated iTTP	Caplacizumab plus immunosuppression without TPE in selected patients	Similar platelet recovery and clinical outcomes; lower ICU use and shorter stay; rescue TPE in non-responders	Early platelet response after first caplacizumab dose is a practical selection tool
Schimpf et al.^17^	Relapsing TTP	Caplacizumab, corticosteroids, and obinutuzumab without TPE	Successful TPE-free management in a single case	Alternative B-cell-directed therapy may be relevant in selected relapsed or rituximab-exposed patients

## 5. Patient Selection: Who May Be Considered?

### 5.1. Confirmed or Highly Suspected Immune-Mediated TTP

Plasma exchange-free management should be considered only when iTTP is confirmed or highly suspected. Because caplacizumab targets VWF-mediated platelet adhesion, it may improve thrombocytopenia in iTTP but does not treat alternative thrombotic microangiopathies such as complement-mediated haemolytic uraemic syndrome, malignant hypertension-associated thrombotic microangiopathy, disseminated intravascular coagulation, drug-induced thrombotic microangiopathy, or cancer-associated microangiopathy.^1,3^ A structured diagnostic assessment using clinical probability tools, haemolysis markers, renal function, coagulation studies, blood film review, and urgent ADAMTS13 testing is essential.^1,3^

In centres where ADAMTS13 testing is delayed, the safety of a plasma exchange-free strategy becomes more uncertain. Standard TPE-based management remains the safest default when the diagnosis is unclear, the patient is unstable, or alternative diagnoses cannot be rapidly excluded.^3,5^

### 5.2. Clinical Stability and Absence of Severe Organ-Threatening Disease

Published experience suggests that neurological involvement alone should not automatically exclude TPE-free treatment. Several reported patients had neurological symptoms and still responded to caplacizumab-based strategies.^5,10,12,15^ The more important distinction is trajectory and severity. Stable or improving symptoms may be compatible with observation after the first caplacizumab dose, whereas rapidly progressive neurological deterioration, coma, seizures, haemodynamic instability, severe cardiac involvement, severe renal impairment, or multiorgan failure should prompt immediate standard therapy including TPE.^3,5^

The clinician should also evaluate comorbidities that may impair platelet recovery or obscure response. In the Kühne cohort, all patients who required rescue TPE had comorbidities or confounders such as active infection, immune thrombocytopenia overlap, viral coinfection, or diagnostic uncertainty.^5^ These factors should be considered relative contraindications to initial TPE omission.

### 5.3. Platelet Response After the First Caplacizumab Dose

The most practical selection criterion is the platelet count trajectory after the first intravenous caplacizumab dose.^5^ A measurable early rise in platelet count, even if modest, suggests that VWF-mediated platelet consumption is being interrupted and supports continued close observation without TPE in an otherwise stable patient. Conversely, failure of the platelet count to increase, continued clinical deterioration, or worsening organ dysfunction should trigger rescue TPE without delay, in addition to initiate additional diagnostic procedures.

This response-guided model is more clinically useful than baseline platelet count alone. Although mild or moderate thrombocytopenia may make clinicians more comfortable with TPE omission, the Kühne cohort included patients with severe thrombocytopenia and still reported favourable outcomes when early response criteria were met.^5^

### 5.4. Situations Where Plasma Exchange-Free Treatment May Be Reasonable

Based on available evidence, plasma exchange-free treatment may be considered in the following settings: refusal of plasma products after informed discussion; severe plasma allergy or anaphylaxis; inability to obtain safe vascular access; early relapsed iTTP in a stable and reliable patient; mild to moderate disease with rapid ADAMTS13 confirmation; and selected patients who demonstrate early platelet response after first intravenous caplacizumab.^5,10-16^ In all cases, the decision should occur in an experienced centre with immediate access to rescue TPE if needed.

### 5.5. Red Flags Requiring Immediate Rescue Plasma Exchange

Red flags include absent platelet response after first caplacizumab dose, falling platelet count despite therapy, haemodynamic instability, rapidly worsening neurological manifestations, seizures or impaired consciousness, severe cardiac involvement, progressive renal failure, diagnostic uncertainty, active uncontrolled infection, suspected alternative thrombotic microangiopathy, or inability to provide close monitoring.^3,5^ In these circumstances, TPE should not be delayed for the sake of preserving a plasma exchange-free strategy.

Table 2 presents a practical framework outlining clinical and institutional criteria to determine which iTTP patients are suitable for TPE-free caplacizumab treatment versus those who require standard plasma exchange.

**Table 2.** Practical Candidate-Selection and Escalation Framework for Plasma Exchange-Free Treatment

Domain	Features supporting consideration	Features arguing against TPE omission
Diagnosis	Confirmed or highly suspected iTTP; urgent ADAMTS13 testing available	Unclear diagnosis; alternative thrombotic microangiopathy likely; delayed or unavailable ADAMTS13 testing
Clinical status	Haemodynamic stability; absent or mild organ dysfunction; stable or improving symptoms	Shock, coma, seizures, rapidly progressive neurological disease, severe cardiac involvement, multiorgan failure
Platelet trajectory	Any early platelet rise after first intravenous caplacizumab dose	No platelet rise, continued fall, or worsening haemolysis/organ injury after first dose
Disease context	Early relapse, plasma refusal, severe plasma reaction, poor venous access, informed patient preference	First presentation with high diagnostic uncertainty or severe disease burden
Comorbidities	No major comorbidity expected to impair platelet response	Active severe infection, immune thrombocytopenia overlap, major confounders, recent platelet transfusion obscuring diagnosis
Infrastructure	Experienced centre, on-site caplacizumab, rapid laboratory support, immediate rescue TPE capacity	No rapid ADAMTS13 testing, no caplacizumab stock, no apheresis backup, unreliable monitoring pathway

## 6. Therapeutic Strategy and Monitoring in Plasma Exchange-Free Management

### 6.1. Caplacizumab Initiation, Early Platelet Response, and Treatment Duration

Caplacizumab should be administered promptly when iTTP is confirmed or strongly suspected and a plasma exchange-free approach is being considered.^4,5^ In this setting, the first intravenous dose has both therapeutic and decision-making value. Therapeutically, it rapidly blocks VWF-mediated platelet adhesion. Clinically, the platelet count response after the first dose helps determine whether it is safe to continue without TPE or whether rescue TPE is required.^5^ After the initial intravenous dose, treatment is continued with daily subcutaneous caplacizumab according to standard practice.^4^ Because this early response guides a high-risk management decision, the first dose should generally be given under inpatient observation. Outpatient or self-administered initiation should be reserved only for highly selected relapse cases managed through an established protocol with reliable monitoring and immediate access to hospital care.^5,14^

The duration of caplacizumab should be guided by ADAMTS13 recovery rather than platelet normalisation alone.^5^ Platelet recovery indicates control of the acute microvascular platelet-consumption process, but it does not prove immunological remission. Continuing caplacizumab until ADAMTS13 activity has recovered to a safe threshold is central to preventing exacerbation after drug discontinuation.^5,7,8^

### 6.2. Immunosuppression: Corticosteroids and Rituximab

Corticosteroids remain a conventional early immunosuppressive component of iTTP treatment.^3^ Although dosing and taper duration vary between centres, their role is to suppress inflammation and reduce autoantibody-mediated disease activity. In plasma exchange-free treatment, corticosteroids should usually be initiated promptly unless contraindicated.

Rituximab is particularly important in this setting because durable remission requires suppression of anti-ADAMTS13 antibody production.^3^ Early rituximab should be strongly considered, especially in relapsed disease, high inhibitor burden, delayed ADAMTS13 recovery, or when TPE is omitted.^5,16^ Alternative B-cell-directed approaches, such as obinutuzumab, remain investigational or case-based but may become relevant in selected patients with rituximab resistance, intolerance, or multiple relapses.^17^

### 6.3. ADAMTS13-Guided Continuation and Discontinuation of Caplacizumab

A safe plasma exchange-free strategy depends on serial ADAMTS13 monitoring.^5^ Weekly ADAMTS13 activity assessment during the acute and early recovery phase is a reasonable minimum standard, with more frequent testing where available or when the clinical course is atypical. Caplacizumab should not be stopped solely because platelet count has normalised. Premature discontinuation before ADAMTS13 recovery leaves the patient exposed to recurrent VWF-mediated platelet thrombosis once the caplacizumab effect wanes.^5,7,8^

### 6.4. Rescue and Adjunctive Strategies When Response Is Inadequate

Rescue or adjunctive treatment should be considered when there is inadequate platelet recovery, clinical deterioration, or persistently delayed ADAMTS13 recovery despite appropriate immunosuppression.^5^ In this context, rescue therapeutic plasma exchange remains the most established escalation strategy when it is feasible and acceptable.

In exceptional circumstances where donor plasma is refused or contraindicated, extracorporeal immunoadsorption may be considered as an adjunct to remove pathogenic antibodies, particularly when ADAMTS13 recovery is delayed despite corticosteroids and rituximab.^12^ ADAMTS13-containing products and recombinant ADAMTS13 are mechanistically attractive because they may partially replace the ADAMTS13-repletion function of plasma exchange without full plasma exchange.^11,18^ However, their role in acquired iTTP remains investigational, and they should not be considered routine substitutes for established therapy outside specialised centres or clinical trials.

**Figure 2.**
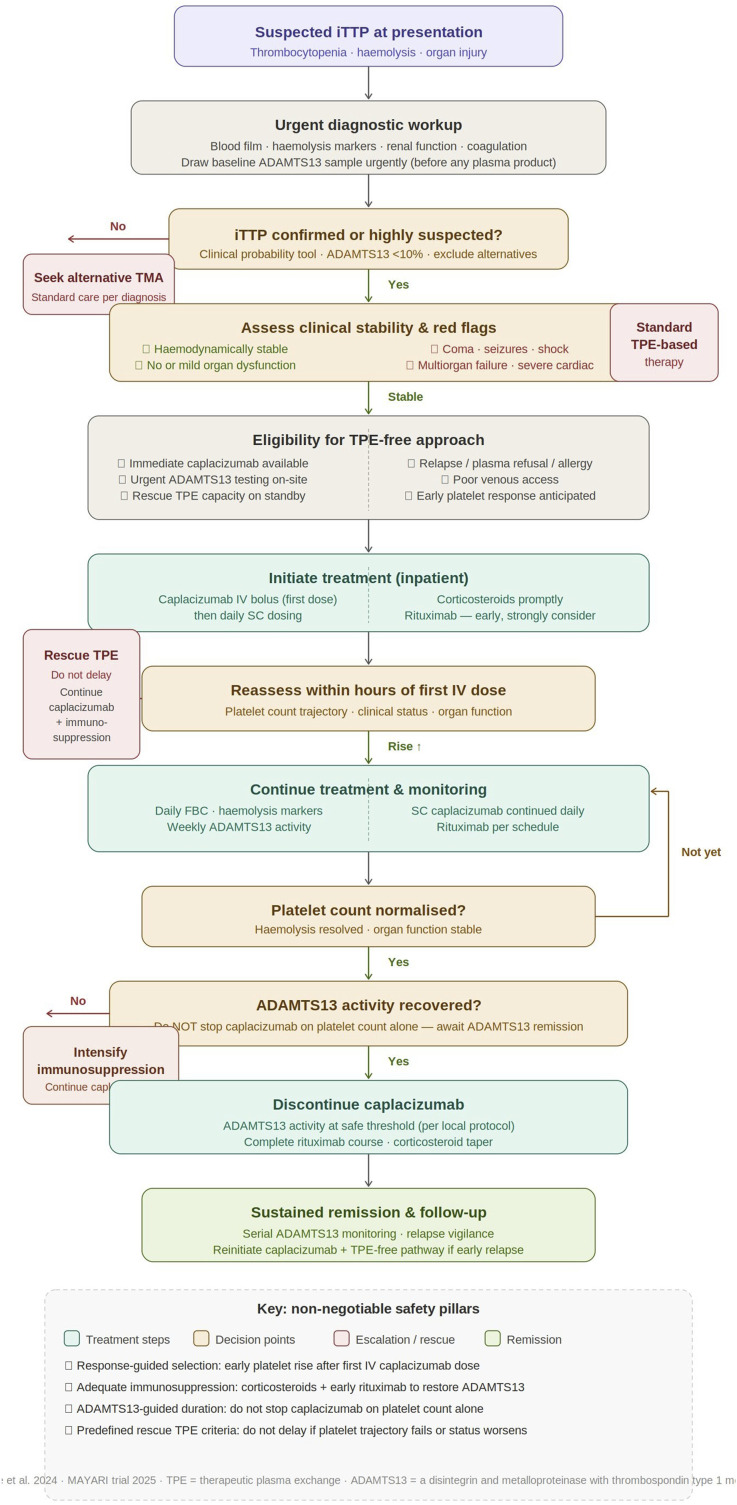
Caplacizumab-based, TPE-free management algorithm for iTTP. Stable patients with confirmed iTTP receive caplacizumab, corticosteroids, and early rituximab; platelet response within hours guides continuation or escalation to rescue TPE. Caplacizumab is discontinued only upon ADAMTS13 recovery, not platelet normalisation alone. TPE = therapeutic plasma exchange; ADAMTS13 = a disintegrin and metalloproteinase with thrombospondin type 1 motif, member 13; iTTP = immune thrombotic thrombocytopenic purpura; SC = subcutaneous; IV = intravenous.

Figure 2 is a proposed caplacizumab-based, TPE-free management algorithm for immune TTP (iTTP). Following urgent diagnostic workup and ADAMTS13 sampling, haemodynamically stable patients without severe red flags are assessed for eligibility and initiated on caplacizumab (IV bolus then daily SC), corticosteroids, and early rituximab. Platelet response within hours of the first dose guides continuation; non-responders proceed immediately to rescue TPE. Treatment continues until platelet normalisation and, critically, ADAMTS13 activity recovery — not platelet count alone — determines caplacizumab discontinuation. Patients are followed with serial ADAMTS13 monitoring for relapse surveillance.

## 7. ADAMTS13 Recovery and Relapse Prevention

### 7.1. Why ADAMTS13 Remission Matters

Clinical remission in iTTP can occur before immunological remission. Platelet count normalisation and haemolysis improvement show that acute microvascular thrombosis has been controlled, but persistent severe ADAMTS13 deficiency indicates ongoing biological vulnerability.^7,8^ Therefore, ADAMTS13 recovery is not merely a laboratory endpoint; it is central to safe caplacizumab discontinuation and long-term relapse prevention.

### 7.2. Does Omitting Plasma Exchange Delay ADAMTS13 Recovery?

A theoretical concern is that omitting TPE could delay ADAMTS13 recovery because TPE removes anti-ADAMTS13 antibodies and provides exogenous ADAMTS13.^2,3^ This concern appeared plausible in some small series where ADAMTS13 recovery was protracted.^16^ However, the largest comparative cohort did not support delayed recovery; in fact, ADAMTS13 recovery to at least 20% occurred faster in the TPE-free group than in the TPE-treated group.^5^ One possible explanation is avoidance of donor plasma exposure and potential ADAMTS13 inhibitor boosting, a phenomenon described in poor responders to plasma exchange.^19^

Registry data also suggest that ADAMTS13 recovery may not be substantially impaired when caplacizumab is used without TPE in selected patients.^20^ Nevertheless, these observations should be interpreted cautiously. Testing schedules were not uniform, treatment selection was not randomised, and patients chosen for TPE-free management may have differed in ways that favoured recovery. Prospective protocol-driven data are needed before firm conclusions can be drawn.

### 7.3. Evidence From Published Cohorts

In the French caplacizumab-era MACHU cohort, standard triple therapy with TPE, caplacizumab, and immunosuppression achieved ADAMTS13 recovery over a timeframe broadly comparable to that later reported in selected TPE-free cohorts.^21^ Prasannan and colleagues highlighted that delayed ADAMTS13 normalisation remains clinically relevant in the caplacizumab era, reinforcing the need to continue monitoring beyond early platelet recovery.^22^ Together, these data support a pragmatic view: the safety of TPE-free treatment is less dependent on whether TPE is used and more dependent on whether ADAMTS13 recovery is actively monitored and acted upon.

### 7.4. Monitoring Strategy and Safe Discontinuation of Caplacizumab

A practical monitoring strategy should include daily blood counts and haemolysis markers during the acute phase, early reassessment after the first caplacizumab dose, and serial ADAMTS13 activity testing.^5^ Caplacizumab discontinuation should be deferred until ADAMTS13 activity has recovered to a prespecified safe range according to local practice or trial protocol. If ADAMTS13 remains severely deficient despite platelet recovery, caplacizumab should generally be continued and immunosuppression intensified or reassessed.^5,7,8^ This approach is particularly important in TPE-free treatment, where the clinician has intentionally omitted both antibody removal and ADAMTS13 replacement.

## 8. Safety Considerations

### 8.1. Bleeding Risk With Caplacizumab

Caplacizumab impairs VWF-mediated platelet adhesion and therefore predictably increases mucocutaneous and procedure-related bleeding risk.^4^ Most bleeding events in caplacizumab trials and real-world studies are manageable, but major bleeding can occur.^4,5^ Importantly, a meta-analysis of 2,956 patients found no statistically significant difference in rates of major bleeding (RR 2.09; 95% CI 0.61–7.14) or major thrombosis (RR 1.06; 95% CI 0.62–1.79) between caplacizumab-treated and control patients.^6^ In the TPE-free literature, clinically important bleeding events included gastrointestinal bleeding and subdural haematoma.^5,16^ These events underscore that the main haemorrhagic risk is related to caplacizumab pharmacology rather than omission of TPE itself.

### 8.2. Concomitant Antiplatelet or Anticoagulant Therapy

Concomitant antiplatelet or anticoagulant therapy requires explicit reassessment. The major gastrointestinal bleeding event reported by Capecchi and colleagues occurred in the context of concurrent aspirin therapy.^16^ In patients receiving caplacizumab, antiplatelet agents and anticoagulants should not be continued reflexively. Their indication, urgency, and bleeding risk should be reviewed, with temporary interruption considered where clinically permissible. Decisions should be individualised, especially in patients with recent coronary stenting, atrial fibrillation, venous thromboembolism, mechanical valves, or other competing thrombotic risks.

### 8.3. Complications Avoided by Omitting Plasma Exchange

Avoiding TPE eliminates several procedural risks, including central venous catheter complications, plasma reactions, citrate toxicity, and the need for repeated apheresis procedures.^2,5^ In the Kühne cohort, serious TPE-related complications occurred in the conventional-treatment group, including severe anaphylactic reactions, whereas these events were avoided in the TPE-free group.^5^ Avoiding TPE may also reduce ICU utilisation and hospital length of stay in selected stable patients.^5^

### 8.4. When the Plasma Exchange-free Approach Becomes Unsafe

The approach becomes unsafe when monitoring is inadequate, diagnosis is uncertain, caplacizumab is delayed or unavailable, ADAMTS13 testing cannot guide therapy, clinical status deteriorates, or rescue TPE cannot be rapidly initiated.^3,5^ It is also unsafe if the absence of TPE is treated as a fixed goal rather than a conditional strategy. The correct operational principle is not to avoid TPE at all costs, but to withhold TPE only while objective response and clinical stability support doing so.

Absence of a robust platelet count increase or atypical platelet count dynamics should not only call clinicians to evaluate the necessity of additional therapeutic plasma exchange, but should also initiate a systematic search for concomitant diseases or confounding conditions that may impair or obscure the expected haematological response. As demonstrated in the largest comparative cohort to date, all patients who ultimately required rescue TPE had identifiable comorbidities or confounders—including active infection, immune thrombocytopenia overlap, viral coinfection, or diagnostic uncertainty—that likely contributed to their atypical platelet trajectories.^5,23^ Recognising these concurrent conditions early is therefore as clinically important as the platelet count response itself, since failure to account for them may lead either to inappropriate escalation of plasma exchange or, conversely, to delayed intervention in a patient who is genuinely failing caplacizumab-based management. This perspective is further supported by a large retrospective multicentre registry study by Kühne and colleagues, which analysed 204 acute iTTP episodes from the German REACT-2020 and Austrian ATMAR registries, all treated with caplacizumab.^23^ In that cohort, 83.8% of patients achieved a clinical response by day 5 after caplacizumab initiation, and only three patients (1.5%) met laboratory criteria for true refractoriness—each of whom had an identifiable confounding factor such as missed doses or active infection. No patient was refractory without such a confounder, and confounding conditions were the sole independent determinant of delayed platelet count normalisation in the stratified Cox model. These findings reinforce that, in the caplacizumab era, true haematological refractoriness of iTTP has become exceedingly rare, and that apparent treatment failure should prompt systematic evaluation for alternative explanations before escalating therapy.^23^

In the absence of confounding factors causing thrombotic microangiopathy or thrombocytopenia through an alternative mechanism, there are essentially only two plausible explanations for apparent caplacizumab failure in a confirmed iTTP patient. The first is a structural variant within the VWF A1 domain that disrupts the nanobody’s binding site, as exemplified by the case reported by Thomas and colleagues describing the first documented instance of caplacizumab resistance mediated by VWF A1 domain missense variants.^24^ The second is non-adherence or missed doses of caplacizumab during the critical early disease course, a scenario that has been discussed in the context of outpatient and self-administration protocols where adherence cannot be directly verified. Both scenarios carry distinct management implications: pharmacogenomic or structural resistance may necessitate a definitive switch to TPE-based rescue with reassessment of future caplacizumab utility, whereas adherence-related failure may be addressable through enhanced monitoring, supervised administration, or inpatient consolidation of therapy. Systematic evaluation of VWF activity levels, which are expected to be significantly suppressed during effective caplacizumab therapy, can serve as a practical biomarker to distinguish true pharmacological failure from adherence-related issues.^24^

## 9. Practical Implementation in Experienced Centres

### 9.1. Minimum Institutional Requirements

Plasma exchange-free management should be restricted to centres with experience in iTTP, immediate caplacizumab availability, urgent laboratory support, reliable ADAMTS13 testing, and capacity for rescue TPE.^5^ Centres without these resources should not attempt this strategy outside a formal referral or trial pathway. The infrastructure requirement is not a minor logistical issue; it is a central safety determinant.

### 9.2. Rapid ADAMTS13 Testing and Laboratory Support

Rapid ADAMTS13 testing is essential for both diagnosis and ongoing treatment decisions.^1,5^ Baseline blood samples should be obtained before plasma-containing products or TPE whenever possible, but treatment should not be delayed in highly suspected iTTP. A TPE-free pathway requires a defined process for ordering, transporting, processing, and reporting ADAMTS13 activity and inhibitor testing, with results integrated into clinical decisions about caplacizumab continuation and immunosuppression.

### 9.3. Immediate Caplacizumab Availability and First-Dose Monitoring

Caplacizumab must be available on site. Delayed access undermines the rationale of a TPE-free strategy because the first hours of treatment are critical for interrupting microvascular thrombosis.^4,5^ After the first intravenous dose, platelet count and clinical status should be reassessed within hours. A favourable early platelet trajectory supports continued observation without TPE, whereas absent response should trigger escalation.^5^

### 9.4. Multidisciplinary Workflow

A practical pathway requires coordination between haematology, transfusion medicine, nephrology or apheresis services, intensive care, emergency medicine, pharmacy, and the haemostasis laboratory.^5^ The pathway should specify diagnostic criteria, first-dose caplacizumab procedures, timing of repeat laboratory tests, criteria for inpatient versus outpatient management, bleeding precautions, ADAMTS13 monitoring intervals, rituximab timing, and rescue TPE triggers.

### 9.5. Suggested Escalation Algorithm

A pragmatic algorithm is as follows. First, identify suspected iTTP using clinical assessment, blood film review, haemolysis markers, platelet count, renal function, coagulation studies, and clinical probability scoring. Second, draw baseline ADAMTS13 samples urgently. Third, administer caplacizumab promptly when iTTP is confirmed or strongly suspected and TPE-free treatment is being considered. Fourth, start corticosteroids and plan early B-cell-directed therapy unless contraindicated. Fifth, reassess platelet count and clinical status within hours of the first intravenous caplacizumab dose. Sixth, continue without TPE only if the patient is clinically stable and demonstrates an early platelet response. Seventh, initiate rescue TPE immediately if platelet response is absent, organ dysfunction progresses, or diagnostic uncertainty increases. Finally, continue caplacizumab until ADAMTS13-guided discontinuation criteria are met.^5^

## 10. Evidence Gaps and Future Directions

### 10.1. Limitations of the Current Evidence

The current evidence base remains limited. Most reports are case reports, small series, or retrospective cohorts. Selection bias is unavoidable because clinicians likely chose TPE-free treatment for patients perceived as more suitable, more stable, or more closely monitorable. Testing schedules, immunosuppressive strategies, ADAMTS13 thresholds, definitions of response, and criteria for rescue TPE vary across studies. Long-term relapse outcomes remain insufficiently characterised.

### 10.2. The Role of the MAYARI Trial

The MAYARI study was designed to prospectively evaluate caplacizumab and immunosuppressive therapy without first-line TPE in adults with iTTP.^25^ Its protocol-driven design is important because it directly addresses whether selected patients can be safely managed without TPE under standardised monitoring and escalation criteria. The trial has now been completed, with primary results presented at the International Society on Thrombosis and Haemostasis (ISTH) Congress in 2025, further supporting the feasibility of caplacizumab-based management without upfront TPE in expert centre settings; full publication is anticipated.^25,26^ These prospective data are expected to shape whether this approach remains limited to exceptional circumstances or becomes an accepted option in predefined patient groups.

### 10.3. Unanswered Questions in Severe Disease

A major unresolved question is whether any subgroup of patients with severe organ-threatening disease can safely avoid TPE. Current evidence supports caution. Patients with coma, rapidly progressive neurological disease, severe cardiac involvement, shock, or multiorgan failure should generally receive standard TPE-based treatment.^3,5^ Plasma exchange-free management should not be extrapolated from stable relapse patients to critically ill first-presentation iTTP without prospective evidence.

### 10.4. Future Role of Recombinant ADAMTS13 and Other Emerging Strategies

Recombinant ADAMTS13 may eventually change the therapeutic landscape by providing targeted ADAMTS13 replacement without plasma exchange.^18^ In principle, combining caplacizumab, immunosuppression, and pharmacological ADAMTS13 replacement could reproduce the main benefits of TPE while avoiding the procedure itself. However, this remains investigational in acquired iTTP. Other unresolved questions include the optimal timing of rituximab, the role of obinutuzumab in selected relapsed or rituximab-resistant patients,^17^ the ideal corticosteroid regimen, cost-effectiveness, and feasibility in resource-limited settings.

## 11. Conclusion

Caplacizumab has created a biologically plausible and increasingly evidence-supported pathway for treating selected patients with iTTP without plasma exchange. The approach is most defensible in experienced centres when iTTP is confirmed or highly suspected, the patient is clinically stable, caplacizumab is immediately available, there is early platelet response after the first intravenous dose, and rescue TPE can be initiated without delay if response is inadequate. However, plasma exchange-free treatment should not yet be framed as a routine replacement for standard TPE-based therapy. The current evidence remains largely observational, and patient selection is central. Successful implementation depends on four non-negotiable principles: response-guided selection using early platelet trajectory, adequate immunosuppression to restore ADAMTS13 activity, ADAMTS13-guided caplacizumab duration, and predefined rescue TPE criteria. Until prospective data mature, this strategy should remain a specialised option for carefully selected patients managed by experienced multidisciplinary teams.

## Data Availability

Not applicable. No new datasets were generated or analyzed during the current study.*

## Appendix

### Abbreviations

ADAMTS13: a disintegrin and metalloproteinase with thrombospondin type 1 motif 13
ICU: intensive care unit
iTTP: immune-mediated thrombotic thrombocytopenic purpura
TPE: therapeutic plasma exchange
TTP: thrombotic thrombocytopenic purpura
VWF: von Willebrand factor
